# Functional Haplotypes of the hTERT Gene, Leukocyte Telomere Length Shortening, and the Risk of Peripheral Arterial Disease

**DOI:** 10.1371/journal.pone.0047029

**Published:** 2012-10-17

**Authors:** Weili Zhang, Yu Chen, Xiaomin Yang, Jingyao Fan, Xuenan Mi, Jizheng Wang, Channa Zhang, Frank B. Hu, Rutai Hui

**Affiliations:** 1 Sino-German Laboratory for Molecular Medicine, the State Key Laboratory of Cardiovascular Disease, FuWai Hospital, National Center for Cardiovascular Disease, Chinese Academy of Medical Sciences and Peking Union Medical College, Beijing, China; 2 The Second Affiliated Hospital of Baotou Medical College, Baotou City, China; 3 Department of Nutrition and Epidemiology, Harvard School of Public Health, Boston, Massachusetts, United States of America; Brigham & Women's Hospital, and Harvard Medical School, United States of America

## Abstract

**Background:**

The development of peripheral arterial disease (PAD) is heterogeneous even in the presence of similar risk factors. Our aim was to determine whether inter-individual differences in leukocyte telomere length contribute to the susceptibility of PAD.

**Methods:**

A total of 485 patients with PAD (defined by the ankle-brachial index) and 970 age- and gender-matched controls were recruited from seven rural communities in Henan Province in China. The relative leukocyte telomere length was determined by a quantitative PCR-based method. Two common promoter variants of the *hTERT* gene were genotyped to assess their effects on telomere length and the risk of PAD. *In vivo* luciferase assay was performed to study the transcriptional activity.

**Results:**

After adjustment for vascular risk factors and genetic variants in the *hTERT* gene, individuals in the lowest and middle tertiles of telomere length had a significantly higher risk of PAD than did those in the highest tertile (odds ratio [OR] 1.73, 95% confidence interval [CI] 1.29–2.49 in the middle tertile; 3.15, 95%CI 2.31–4.29 in the lowest tertile). Haplotype analysis using the 2 variants (rs2735940 and rs2853669) showed that subjects with the at-risk C-C haplotype had shorter telomere length than those individuals with the T-T haplotype and consistently had 1.30-fold (OR 1.30, 95%CI 1.06–1.58; *P* = 0.005) increased risk for PAD. The C-C haplotype had 43% lowered transcription activity of *hTERT* promoter (*P*<0.001).

**Conclusion:**

The associations between the functional haplotype of *hTERT* gene and telomere length and the risk of atherosclerotic PAD suggested that mean leukocyte telomere length may independently serve as a potential predictor of PAD.

## Introduction

Atherosclerotic peripheral arterial disease (PAD) is a disease characterized by progressive narrowing of arteries in the lower limbs, and patients often suffer from leg ischemia, intermittent claudication, and reduced quality of life. Importantly, both asymptomatic and symptomatic PAD are associated with increased risk of stroke, myocardial infarction, and death [1]–[4]. PAD affects approximately 8–10 million people in the United States [5] and is increasingly prevalent in Europe and Asia [6]–[8]. In China, the prevalence of PAD was 8.7% among hypertensive patients in our previous study [9]. PAD patients have vascular risk factors similar to that of coronary artery disease, whereas at an individual level, the progression of PAD seems to be highly variable even in the presence of the similar risk factors profiles. The inter-individual heterogeneities are still incompletely understood, but biological aging is one of the contributors.

Telomere attrition represents one molecular mechanism that contributes to cellular aging. Telomeres are specialized DNA-protein structures at the end of all chromosomes, which preserve chromosome stability and integrity [10]. During somatic cell division, DNA polymerase cannot fully replicate the 3′ end of linear DNA, resulting in a progressive loss of telomeres. When telomere lengths are shortened to a critical value, they lose capping function at the chromosomal ends, activate the DNA damage checkpoints, and eventually result in cellular senescence/apoptosis which has been linked with the pathogenesis of atherosclerosis [11]. Synthesis and maintenance of telomeres are mediated by a specialized ribonucleoprotein complex known as telomerase. Human telomerase catalytic subunit, encoded by telomerase reverse transcriptase (*hTERT*) gene, has shown to be a rate-limiting determinant of telomerase activity and maintains genomic stability by adding the telomere repeat TTAGGG to telomere ends [12]–[14]. Recent experimental studies suggested a protective effect of hTERT against endothelial dysfunction [11], [15], [16].

Leukocyte telomere length is inheritable and there is considerable inter-individual variation among people of the similar age [17]. Its heritability has been estimated to be between 35% and 80% on the basis of twin and family studies [18]–[20]. A number of suggestive genetic loci for telomere length in humans have been mapped to chromosome 3p26.1, 10q26.13, 12q12.22, 14q23.2 in family-based linkage studies [19]–[21] and to chromosome 18q12.2 [22], 3q26 [23], [24], [25], 10q24.33 [25], and 14q21 [26] in recent genome-wide association analyses; but these identified loci, except for *TERC* gene, are not replicated in multiple independent studies and only explain a small fraction of the heritability of telomere length. And none of the identified variants have been associated with cardiovascular disease [27]. In contrast, functional variants in *hTERT* gene have been reported to be associated with telomere length and the risk of coronary heart disease in some studies although not in others [28]–[30].

Emerging studies have provided consistent evidence that telomere length shortening is a potential risk predictor for coronary heart disease [31], [32]; however, despite the prevalence and high societal burden of PAD, our understanding of the genetic basis of PAD is still limited [33], [34]. Therefore, in the current community-based case-control study, we aimed to investigate whether mean leukocyte telomere length is a predictor of the development of atherosclerotic PAD, and evaluate the effect of common variants in the *hTERT* gene on risk difference of PAD.

## Methods

### Ethics Statement

The study was complied with the Declaration of Helsinki and was approved by Institutional Review Board and Ethics Committee of Fu Wai Hospital. All participants reported themselves as Han nationality, and provided written informed consent.

### Study Population

This community-based case-control study consisted of 485 patients with PAD and 970 control subjects who were from seven rural communities at the XinYang Country in the middle region in China from 2004 to 2005 [9]. PAD was diagnosed when patients had typical symptoms of intermittent claudication, such as cramping pain of the calves or buttocks during exercise, or an ankle-brachial index (ABI) of ≤0.9 in either leg, calculated according to the recommendations of the American Heart Association [35]. The ABI was determined from Doppler-derived measurements of systolic blood pressure at the brachial and ankle arteries (detailed description shown in the File S1). An ABI >0.9 and ≤1.4 in both legs was considered normal. Subjects were excluded when they had any known diseases including heart failure, valvular heart disease, secondary hypertension, and severe debilitating chronic illness (cancer, renal, or hepatic diseases). For each case, 2 control subjects matched by age (±5 years) and gender and without PAD were recruited from the same communities.

Each participant was interviewed in a community clinic and completed a standardized questionnaire that included demographic factors, medical history, lifestyle, and familial history. Anthropometric measurements, including height, weight, waist and hip circumference (measured at the umbilicus and the widest point, respectively), were measured by trained researchers. Electrocardiogram was performed in all participants. Hypertension was defined as systolic blood pressure of ≥140 mmHg or diastolic blood pressure of ≥90 mmHg or currently taking medication for hypertension.

Fasting blood was drawn from an antecubital vein after overnight. Serum was separated on-site, then transported on dry ice to Beijing center laboratory, and stored at −70°C until measurement. Biochemical variables, including blood glucose, total cholesterol, triglycerides, high-density lipoprotein (HDL) cholesterol, and uric acid were assayed by an automatic analyzer (Hitachi 7060, Tokyo, Japan) in the core laboratory at FuWai Hospital.

### Leukocyte Telomere Length Assay

Genomic DNA was isolated from peripheral blood leukocytes according to standard procedures. Relative mean leukocyte telomere length was determined with a quantitative real-time polymerase chain reaction (PCR)-based technique that compares telomere repeat copy number (T) to single-copy gene copy number (S) (T/S ratio) in a given sample [36]. All PCRs were performed on the Bio-Rad DNA Engine Opticon 2 Real-time PCR Detector (Bio-Rad Ltd, Hercules, CA, USA).

In brief, two master mixes of PCR reagents were prepared, one for the telomere reaction and one for the single-copy gene reaction (*β-globin* gene on chromosome 11p15.5). The primer sequences and thermal cycling profiles were given in details in the File S1. All samples for both the telomere and single-copy gene amplifications were done in duplicate in 96-well plates. The human embryonic kidney 293 (HEK293S) cell line was used as standards for the measurement of mean telomere length. A dilution series (1.56 to 100.00 ng; 2-fold dilution; 7 points) using genomic DNA derived from the HEK293S cell line were included with each 96-well plate for the telomere and the *β-globin* PCRs. The 25-ng standard curve point was used as the reference sample. The slope of the standard curve for the telomere and *β*-globin reactions was −0.22 and −0.35, respectively, and the linear correlation coefficient (*R* ^2^) value for both reactions were >0.99 (File S3). The average inter-plate coefficient of variability was 6.6% for telomere assays and 4.8% for *β-globin* assays. As part of routine quality control, 10% of the samples were randomly chosen to test the reproducibility of the assay. All measurements were performed by technicians blinded to the case-control status.

### *hTERT* Gene Variants Selection and Genotyping

Telomere length is regulated by telomerase activity, and the expression level of human telomerase reverse transcripts (hTERT) is a major determinant of telomerase activity. The *hTERT* gene is located on chromosome 5p15.33 (geneID: 7015), which is strictly regulated by the transcriptional activity of the promoter region. We sequenced the promoter region of the *hTERT* gene (Genbank accession no. AF098956) [37] in 50 randomly chosed control subjects and identified three common variants with a minor allele frequency above 10%, rs2736109 (−1600 G/A), rs2735940 (−1327T/C), and rs2853669 (−190T/C) with positions defined by the transcription initiation site as +1 [37] (File S3). By searching putative nuclear factor-binding sites, we found that rs2853669 is located at a binding site for the transcriptional factor Ets2, which is a critical regulator of the *hTERT* gene expression [37]–[39]. The variant rs2735940 (−1327T/C) has been reported to be associated with coronary artery disease in some studies but not replicated in others [27]–[29]. Based on the potential biological functions, we selected the 2 tagging variants rs2853669 and rs2735940 for further analysis in this association study.

The 2 selected variants were genotyped using the polymerase chain reaction-restriction fragment length polymorphism approach without knowledge of case or control status (Primers was shown in File S2). Reproducibility of genotyping was confirmed by sequencing in 400 randomly selected samples with 100% concordance.

### Construction of *hTERT* Promoter Plasmids and Luciferase Reporter Assays

To determine whether the genetic variants and their haplotypes affect the transcription activity of *hTERT* gene, a series of luciferase reporter plasmids were constructed. First, because rs2853669 resides within a binding site for the transcriptional factor Ets2 and partly overlapped with the transcriptional factor c-Myc, which regulates the *hTERT* gene expression [38], we generated 2 types of luciferase reporter plasmids containing rs2853669TT- or CC- genotype: pGL3-rs2853669T and pGL3-rs2853669C. Second, we generated 4 types of luciferase reporter plasmids with different haplotypes: pGL3-TT (T-T haplotype with rs2735940T and rs2853669T), pGL3-TC (T-C haplotype with rs2735940T and rs2853669C), pGL3-TT (C-T haplotype with rs2735940C and rs2853669T), and pGL3-CC (C-C haplotype with rs2735940C and rs2853669C). The 1.5-kb fragment of the proximal promoter of *hTERT* gene (Genbank accession no. AF098956), encompassing rs2735940 and rs2853669, was amplified and cloned into the pGL3-basic vector (Promega, Madison, WI, USA). The pGL3-basic vector contains the cDNA encoding firefly luciferase; when it was fused with a promoter and transfected into mammalian cells, the construct can be used to analyze the inserted promoter activity. In addition, we constructed plasmids for expression of pcDNA3.1-Ets2 and pcDNA3.1-c-Myc, and investigated whether the transcription factors Ets-2 and c-Myc modify the effects of genetic variants on the transcription activity of *hTERT* gene. Primers for constructing the reporter plasmids were shown in File S2. All the constructs were confirmed by sequencing.

Hela cells and Hek293S cells were seeded in 96-well plates, respectively. On the day of transfection, each well was co-transfected with 0.2 µg of the pGL3 vector, 5 ng of the pRL-TK vector (Promega), 0.1 µg of pcDNA3.1-Ets2, or pcDNA3.1-c-Myc by using Lipofectamine 2000 (Invitrogen, Carlsbad, CA, USA). The pRL-TK vector, encoding the Renilla luciferase, was used as internal control to normalized firefly luciferase expression. Twenty-four hours after transfection, the cell lysates were prepared with the Dual-luciferase reporter assay system (Promega), and *Firefly* and *Renilla* luciferase activities were measured with a luminometer (Turner, Sunnyvale, CA, USA). The transfection efficiency was normalized according to the *Renilla* luciferase activity. Each transfection was carried out in triplicate and repeated 3 times.

### Statistical Analysis

Normal distribution of data was examined by the Kolmogorov–Smirnov normality test. The T/S ratios of mean leukocyte telomere length were natural logarithm transformed because of a skewed distribution. Characteristics of cases and controls were compared by χ^2^ test for categorical variables and two-sample *t* test for quantitative variables. The distributions of the T/S ratio of telomere length were divided into tertiles among control subjects, and the cutoff values were <1.87 for the lowest tertile, 1.87–3.23 for the middle tertile, and >3.23 for the highest tertile. The odds ratios (ORs) and 95% confidence intervals (CIs) were estimated for the association between telomeres and the risk of PAD by using conditional logistic regression models. Multivariate analyses were adjusted for body mass index, fasting triglycerides, total cholesterol, high-density lipoprotein cholesterol (HDL-C), fasting blood glucose, blood pressure, smoking status (never, past, current), alcohol intake (current drinker, yes/no), and further for medical history, including hypertension (yes/no), diabetes (yes/no), previous cardiovascular disease (yes/no), and antihypertensive therapy (yes/no).

The χ^2^ test was used to examine the Hardy-Weinberg equilibrium for each variant and to compare the distribution of allele and genotype frequencies between cases and control subjects. Generalized linear regression model was used to compare the differences in telomere length across the genotypes by adjustment for the covariates mentioned above. When the overall difference was statistically significant, a Tukey test was performed as post hoc test to identify significant differences between the 3 genotype groups. The associations between the *hTERT* gene variants and risk of PAD were estimated using conditional logistic regression analysis by adjustment for the covariates mentioned above, and the *P* value was corrected for multiple comparisons (for the 2 tested variants and the 3 genetic models) by Simes' method, a modified Bonferroni procedure [40]. The linkage disequilibrium between tested variants was calculated using Haploview software 4.2, and the R^2^ was used to indicate the strength of linkage disequilibrium. Haplotype analysis was conducted on the basis of the Stochastic-EM algorithm using the THESIAS program [41]. The sex-specific associations were also examined by computing the interaction term.

The population-attributable fraction was estimated for variants with the following equation: population-attributable fraction% = 100×p(OR−1)÷[p(OR−1)+1]; p is the frequency of the at-risk genotypes among control subjects. All probability values were 2-sided, and *P*<0.05 was considered significant. Analyses were performed with SPSS software, version 13.0 (SPSS Inc, Chicago, USA).

## Results

### Clinical Characteristics of Study Participants

In this study, age, presented as mean±SD, was 58.6±9.4 years in cases and 58.3±9.4 years in controls, and men accounted for 36.9% of cases and 38.3% of controls (Table 1). As expected, PAD patients had a higher prevalence of history of cardiovascular disease and hypertension; they also had higher levels of systolic blood pressure and total cholesterol. There were no significant differences in clinical characteristics by tertiles of mean telomere length in PAD patients and control subjects, including blood pressure, smoking, alcohol intake, and medical treatments of antihypertensive therapy, blood-glucose control and lipid-lowing therapy (File S2).

**Table 1:** Characteristics of peripheral arterial disease patients and control subjects.*

Characteristics	Cases with PAD (n = 485)	Control subjects (n = 970)	*P* value†
Age, years	58.6±9.4	58.3±9.4	0.52
Male, n (%)	179 (36.9%)	372 (38.3%)	0.60
Body mass index, kg/m^2^	25.2±3.7	25.7±3.6	0.07
Waist-hip ratio	0.87±0.06	0.88±0.06	0.28
Systolic blood pressure, mm Hg	163±29	158±28	0.002
Diastolic blood pressure, mm Hg	96±14	95±13	0.10
Glucose, mmol/L	5.62±2.19	5.49±1.88	0.21
Lipids, mmol/L			
Total cholesterol	5.59±1.27	5.46±1.13	0.05
Triglycerides	1.39 (0.96–1.91)	1.31 (0.95–1.80)	0.28
HDL cholesterol	1.56±0.33	1.55±0.35	0.35
LDL cholesterol	3.17±0.99	3.10±0.90	0.15
Serum creatinine, µmol/L	67.9±38.0	66.7±31.4	0.52
Cigarette smoking, n (%)	105 (21.6%)	178 (18.3%)	0.13
Alcohol intake, n (%)	114 (23.5%)	194 (20.0%)	0.12
Medical history, n (%)			
Hypertension	346 (71.3%)	614 (63.2%)	0.002
Diabetes mellitus	21 (4.3%)	35 (3.6%)	0.50
Cardiovascular disease	103 (21.2%)	31 (3.2%)	<0.0001
Medication treatment, n (%)			
Antihypertension	294 (85.0%)	507 (82.4%)	0.31
Blood-glucose control, n (%)	18 (85.7%)	25 (69.4%)	0.17
Lipid-lowing therapy	52 (65.8%)	91 (65.5%)	0.96
Telomere length			
Relative T/S ratio	1.88±1.00	2.76±1.51	<0.0001

PAD indicates peripheral arterial disease; HDL, high-density lipoprotein cholesterol; LDL, low-density lipoprotein cholesterol; T, telomere repeat copy; S, single-copy gene *globin* copy.

* Data are given as mean ± SD, numbers (percentage) or medians (interquartile range). Telomere length is expressed as a relative telomere/single-copy gene (T/S) ratio.

† *P* value was calculated between PAD patients and control subjects by the two-sample *t*-test for comparison of continuous variables, the χ^2^ test for categorical variables, and the Mann-Whitney U test for triglycerides and telomere length.

### Association between Leukocyte Telomere Length and PAD

The mean leukocyte telomere length was significantly shorter in PAD patients than in control subjects (mean ±SD: 1.88±1.00 versus 2.76±1.51; *P*<0.001; File S3). Overall, the telomere T/S ratio (natural log-transformed) was significantly inversely correlated with chronological age, and decreased 8% per decade (95% CI −3.2 to −13.4; correlation coefficient γ = −0.10; *P*<0.001) in control subjects and 6% per decade (95% CI −3.0 to −13.8; correlation coefficient γ = −0.11, *P*<0.001) in PAD patients, respectively (File S3). However, there was no difference in the regression line slopes between controls and cases (*P* = 0.42).

In both crude analysis and multivariable analysis adjustment for conventional vascular risk factors, individuals in the lowest and middle tertiles of telomere length had a significantly higher risk of PAD than did those in the highest tertile (multivariate OR 1.71, 95%CI 1.24–2.36 in the middle tertile; 3.12, 95%CI 2.32–4.18 in the lowest tertile; *P* for trend<0.0001); and further adjustment for the effect of genetic variants in the *hTERT* gene, the association between shorter telomere lengths and increased risk of PAD was not remarkably changed (Table 2). The associations were not observed a sex-specific effect. In subsidiary analyses, telomere length was analyzed as a continuous variable and was associated with 75% increased risk of developing PAD per 1-SD decrease (multivariate OR 1.75, 95%CI 1.55–1.97; *P*<0.0001). We also tested for the effect modification by age, gender, smoking, alcohol intake, history of hypertension, medication treatment, and genetic variants by performing analyses stratified by these variables and by evaluating interaction terms. Decrease of leukocyte telomere length remained to be associated with the risk of PAD in various groups (File S3). Because the observed association between shorter telomeres and PAD could be biased due to the association with coronary heart disease, we also performed sensitivity analyses by excluding those subjects with history of heart diseases, and the results were not substantially changed (File S2). Of the risk factors measured, marginally significant inverse associations were found between telomere length and waist-hip ratio, serum total cholesterol, and LDL cholesterol, which indicated that longer telomeres corresponded with a better health status (File S2).

**Table 2:** Risk of peripheral arterial disease in different tertiles of leukocyte mean telomere length.

	In tertile groups of relative T/S ratio				Per 1-SD decrease in ln-transformed relative T/S ratio	
	Highest tertile (>3.23)	Middle tertile (1.87–3.23)	Lowest tertile (<1.87)	*P* for rend		*P*
Cases with PAD (n = 485)	49 (10.1%)	147 (30.3%	289 (59.6%)			
Control subjects (n = 970)	324 (33.4%)	324 (33.5%)	322 (33.2%)			
Odds ratio (95%CI)*						
Crude model	1.0	1.90 (1.37–2.58)	3.32 (2.47–4.47)	<0.0001	1.78 (1.58–2.01)	<0.0001
Multivariable model I†	1.0	1.82 (1.31–2.55)	3.22 (2.36–4.38)	<0.0001	1.77 (1.57–1.99)	<0.0001
Multivariable model II‡	1.0	1.71 (1.24–2.36)	3.12 (2.32–4.18)	<0.0001	1.75 (1.55–1.97)	<0.0001
Multivariable model III§	1.0	1.73 (1.29–2.49)	3.15 (2.31–4.29)	<0.0001	1.74 (1.54–1.97)	<0.0001

PAD indicates peripheral arterial disease; CI, confidence interval.

* Odds ratio and 95%CI were obtained with multivariate conditional logistic regression analysis.

† Model I: Adjustment for body mass index, systolic and diastolic blood pressure, smoking, alcohol intake, fasting glucose, triglycerides, total cholesterol, HDL cholesterol, and LDL cholesterol.

‡ Model II: Adjustment for the covariates mentioned above plus diabetes, history of hypertension, previous cardiovascular disease, and medication treatment.

§ Model III: Adjustment for individual genetic variants rs2735940 and rs2853669 of the *hTERT* gene, except for the covariates mentioned in Model II.

### *hTERT* Gene Variants Predispose to Telomere Length Shortening and PAD Risk

The frequencies of rs2735940 and rs2853669 in the *hTERT* gene did not deviate significantly from Hardy-Weinberg equilibrium in this case-control sample population (all *P*>0.05). Before implementation of this study, we performed a statistical power analysis to verify whether the recruited sample could provide adequate power to identify the genetic association [42]. Based on the present sample size, assuming 80% statistical power, at an alpha of 0.05, we had the ability to detect an association with ORs of ≥1.10 if the minor allele frequency is 0.50 and ≥1.90 if the minor allele frequency is 0.01, assuming an additive model.

In an allelic association analysis, the presence of the C-allele of rs2853669 was associated with increased risk of PAD (Table 3). After adjustment for body mass index, vascular risk factors, and telomere lengths, subjects with the CC genotype of rs2853669 had higher susceptibility to PAD compared with the wild-type TT genotype carriers (additive model: OR 1.27, 95%CI 1.08–1.51, *P* = 0.005; dominant model: OR 1.35, 95%CI 1.06–1.71, *P* = 0.005; Table 3). The estimated population-attributable fraction for PAD was 8.9% for the at-risk genotype of CC. After correction for multiple testing by Simes' procedure, the associations remained statistically significant. Because hypertension is an important risk factor for PAD, an additional analysis in the hypertensive patients was conducted, and our data showed that these positive associations were independent of the status of hypertension. For the variant rs2735940, we did not observe the association with PAD after adjustment for vascular risk factors and further for multiple testing.

**Table 3:** Association between variants in the *TERT* gene and risk of peripheral arterial disease.

Genetic variants	PAD cases (n = 485)	Controls (n = 970)	Crude OR (95%CI)*	Adjusted OR (95%CI) †	Adjusted *P* †	Corrected *P* ‡
rs2735940 (−1327T>C)						
Allele						
T	57.6%	60.1%				
C	42.4%	39.9%				
Allelic Association			1.11 (0.95–1.30)	1.08 (0.90–1.26)	0.21	0.56
Genotype, n (%)						
TT	160 (33.0%)	322 (33.3%)	Ref.	Ref.		
TC	239 (49.3%)	522 (53.8%)	0.92 (0.72–1.18)	0.87 (0.68–1.12)	0.29	0.58
CC	86 (17.7%)	126 (13.0%)	1.38 (0.99–1.92)	1.27 (0.90–1.79)	0.17	0.51
Additive model (CC *vs.*TC *vs.*TT)			1.12 (0.95–1.32)	1.08 (0.91–1.28)	0.37	0.74
Dominant model (CC+TC *vs.*TT)			1.01 (0.80–1.28)	0.97 (0.76–1.23)	0.78	0.78
Recessive model (CC *vs.* TC+TT)			1.44 (1.06–1.94)	1.38 (1.01–1.88)	0.04	0.24
rs2853669 (−190T>C)						
Allele						
T	56.7%	62.5%				
C	43.3%	37.5%				
Allelic Association			1.27 (1.09–1.49)	1.21 (1.02–1.40)	0.002	0.02
Genotype, n (%)						
TT	149 (30.7%)	367 (37.8%)	Ref.	Ref.		
TC	252 (52.0%)	478 (49.3%)	1.30 (1.02–1.65)	1.27 (0.99–1.64)	0.06	0.36
CC	84 (17.3%)	125 (12.9%)	1.66 (1.18–2.32)	1.62 (1.14–2.29)	0.006	0.05
Additive model (CC *vs.*TC *vs.*TT)			1.29 (1.10–1.52)	1.27 (1.08–1.51)	0.005	0.04
Dominant model (CC+TC *vs.*TT)			1.37 (1.09–1.73)	1.35 (1.06–1.71)	0.005	0.04
Recessive model (CC *vs.* TC+TT)			1.42 (1.03–1.90)	1.38 (1.01–1.89)	0.04	0.16

PAD indicates peripheral arterial disease; OR, odds ratio.

* Crude ORs (95%CI) were determined by χ^2^ test, cases vs. control subjects.

† Adjusted ORs (95%CI) and adjusted *P* value were obtained with multivariate conditional logistic regression analysis by adjusting for body mass index, triglycerides, total cholesterol, HDL cholesterol, LDL cholesterol, blood glucose, blood pressure, smoking, alcohol intake, diabetes, history of hypertension and cardiovascular disease, medication treatment, and telomere lengths.

‡ Corrected *P* value was obtained by the Simes' procedure, a modified Bonferroni correction for multiple comparisons.

**Figure 1:**
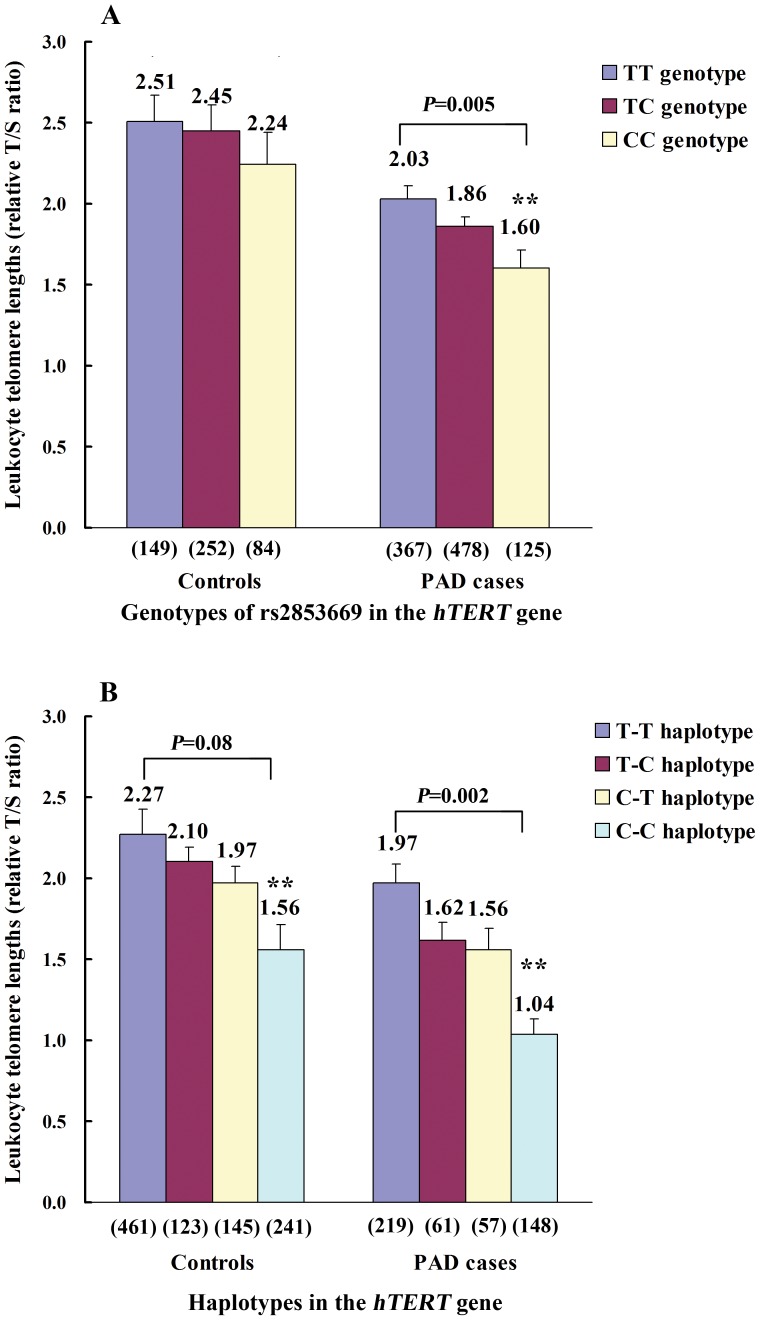
Correlations between telomere length and *hTERT* gene variants. Data shown were the means ± S.E.M. S.E.M. denotes standard error of the mean. Multiple linear regression analysis was used to compare the mean leukocyte telomere lengths by genotypes of rs2853669 (Panel A) or by haplotypes containing rs2735940 and rs2853669 (Panel B) in the *hTERT* gene promoter region among PAD patients and control subjects, respectively, after adjustment for age, gender, and conventional vascular risk factors. Haplotype analysis was conducted on the basis of the Stochastic-EM algorithm using THESIAS program, and possesses 2 loci rs2735940 and rs2853669 from left to right. **P = 0.005, compared with wild-type TT genotype; *P* = 0.002 and *P* = 0.08, compared with wild-type T-T haplotype.

Figure 1A showed the mean leukocyte telomere length by genotypes of rs2853669 in the *hTERT* gene. After adjustment for age, gender, and other vascular risk factors with multiple linear regression analysis, telomere length was correlated to rs2853669 in PAD patients, but not the controls, and the standardized coefficient β for the at-risk C-allele of rs2853669 was −0.17 (*P* = 0.005) compared to the wild-type T-allele. The mean leukocyte telomere length was markedly shorter in carriers with the CC genotype of rs2853669 than that in the TT carriers among PAD patients (1.60±0.11 *vs.* 2.03±0.08; *P* = 0.005; Figure 1A). No correlations were found between telomere length and the variant rs2735940 (File S2).

In addition, given that the effect of a single gene or variant on a complex disease is expected to be modest, a haplotype analysis using the 2 variants (rs2735940 and rs2853669) was performed to assess the haplotype effect on telomere length shortening and the risk of PAD. Subjects with the at-risk C-C haplotype had shorter telomere length than those individuals with the wild-type T-T haplotype among PAD patients (1.04±0.09 *vs.* 1.97±0.12; *P* = 0.002), while a marginally significant difference was found among the controls (Figure 1B). Consistently, the C-C haplotype was associated with 1.30-fold (OR 1.30, 95%CI 1.06–1.58; *P* = 0.005) increased risk of PAD after adjustment for vascular risk factors and telomere lengths (Table 4).

**Table 4:** Haplotype analysis of variants rs2735940 and rs2853669 and the risk of peripheral arterial disease.

	Controls	Cases	Crude ORs	Multivariable model I†		Multivariable model II‡	
Haplotypes*	(n = 970)	(n = 485)	(95% CI)	ORs (95% CI)	*P*	ORs (95% CI)	*P*
T-T	0.477	0.446	Ref.	Ref.		Ref.	
T-C	0.124	0.130	1.08 (0.83–1.39)	1.10 (0.84–1.43)	0.49	1.06 (0.80–1.34)	0.43
C-T	0.148	0.122	0.84 (0.65–1.09)	0.84 (0.64–1.10)	0.20	0.82 (0.62–1.05)	0.18
C-C	0.251	0.302	1.33 (1.09–1.61)	1.36 (1.12–1.66)	0.002	1.30 (1.06–1.58)	0.005
Global *P*					0.003		0.004

* Haplotype analysis was conducted on the basis of the Stochastic-EM algorithm using THESIAS program. Haplotypes possess 2 loci rs2735940 and rs2853669 from left to right.

† Multivariable model I: Adjustment for conventional risk factors, including body mass index, triglycerides, total cholesterol, HDL cholesterol, blood glucose, blood pressure, smoking, alcohol intake, diabetes, history of hypertension, and medication treatment.

‡ Multivariable model II: Adjustment for telomere lengths, except for those covariates mentioned in model I.

### *hTERT* promoter with C-C haplotype had lower transcription activity

**Figure 2:**
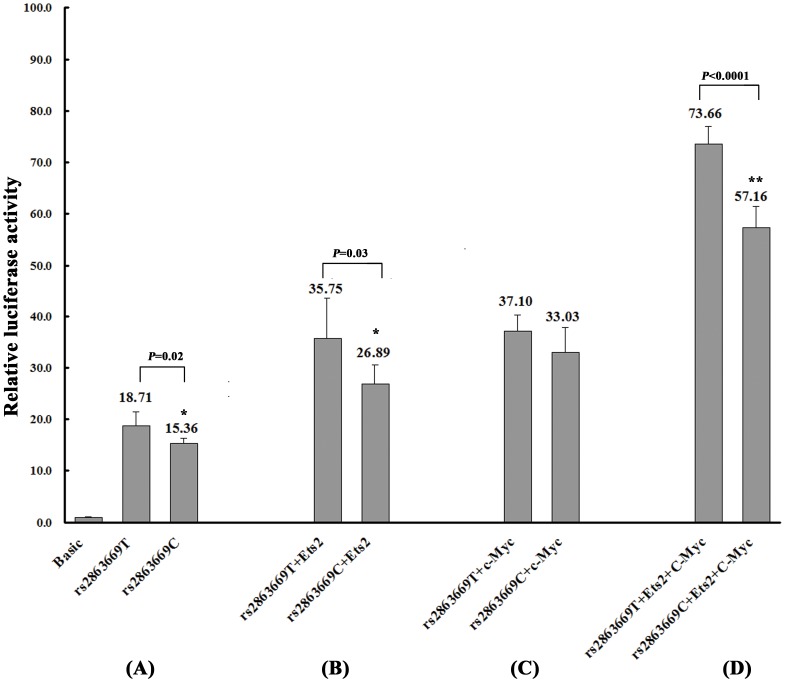
Effect of individual variant rs2853669 on the promoter transcription activity of *hTERT* gene. The luciferase reporter plasmids containing the *hTERT* gene promoter region with rs2853669TT- or CC- genotype were constructed and then transfected into Hek293S cells. The pGL3-Basic is the promoter-less vector as a negative control. Firefly luciferase activity was expressed relative to control, normalized to Renilla luciferase activity to correct for transfection efficiency. (A) Without co-transfection of transcription factors Ets2 and c-Myc; (B) With co-transfection of Ets2; (C) With co-transfection of c-Myc; (D) With co-transfection of both Ets2 and c-Myc. Data shown are the means ± SD, n = 3. *P = 0.02, *P* = 0.03, **P<0.001, compared with the relative luciferase activity of the wild-type rs2853669T.

To investigate whether the individual variants and haplotypes affect the transcription activity of *hTERT* gene, we constructed a series of luciferase reporter plasmids containing rs2735940 and rs2853669 and measured the luciferase activity representative of *hTERT* promoter activity. The results showed that the rs2863669CC genotype has 17% decreased luciferase activity than the wild-type genotype of rs2863669TT (*P* = 0.02, Figure 2). When co-transfected with the transcriptional factors Ets2 and c-Myc in *in vivo* luciferase assays, Ets2 and c-Myc had a combined effect by enhancing the promoter activity of *hTERT* gene, and the promoter activity of the rs2863669CC genotype decreased 22% (Figure 2).

**Figure 3:**
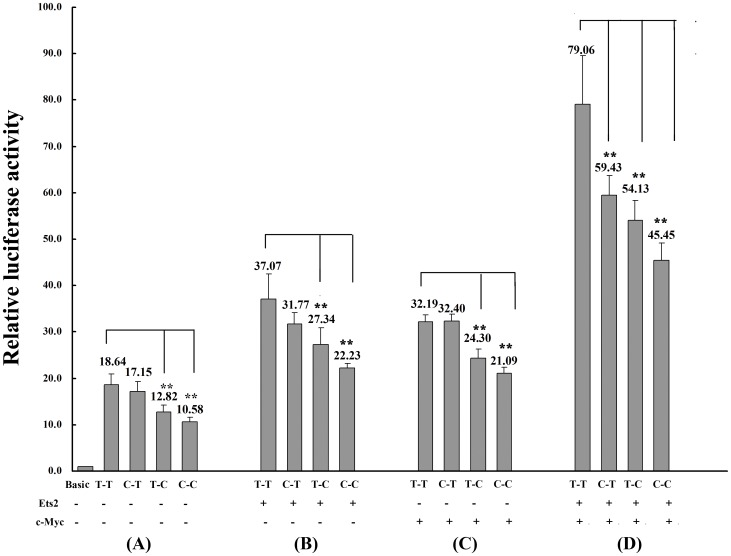
Haplotype effect of variants rs2735940 and rs2853669 on the promoter transcription activity of *hTERT* gene. The luciferase reporter plasmids containing the *hTERT* gene promoter region with haplotype T-T, T-C, C-T, or C-C (rs2735940 and rs2853669) were constructed and then transfected into Hek293S cells. (A) Without co-transfection of transcription factors Ets2 and c-Myc; (B) With co-transfection of Ets2; (C) With co-transfection of c-Myc; (D) With co-transfection of both Ets2 and c-Myc. Data shown are the means ± SD, n = 3. **P<0.001, compared with the relative luciferase activity of the wild-type haplotype T-T.

Compared with the wild-type T-T haplotype, the *hTERT* promoter haplotype carrying the at-risk rs2853669C allele had significantly lower luciferase activity, with 31% decrease for the T-C haplotype and 43% decrease for the C-C haplotype (*P*<0.001), respectively; whereas no significant difference was detected for the C-T haplotype with no stimuli of transcriptional factors (Figure 3). When co-transfected with the transcriptional factors Ets2 and c-Myc in *in vivo* luciferase assays, a combined effect on the promoter activity of *hTERT* gene was observed, and the transcription activity of the C-T, T-C or C-C haplotypes decreased significantly compared with the wild-type T-T haplotype (Figure 3). Results in Hela cells were consistent with that in Hek293S cells (data not shown).

## Discussion

In the present study, to our knowledge, our data for the first time showed that leukocyte telomere length shortening is significantly associated with increased risk for atherosclerotic PAD. We also found that a loss-of-function haplotype C-C (rs2735940 and rs2853669) in the promoter region of the *hTERT* gene (a major determinant of telomerase activity), affects the transcriptional power in *vivo* luciferase activity assays and is associated with shorter telomere length in patients with atherosclerotic PAD. In addition, our results showed that carriers with the at-risk C-C haplotype have increased risk for the development of PAD. The associations were independent of those conventional vascular risk factors.

Atherosclerotic PAD, a common manifestation of systematic atherosclerosis, is closely linked to the aging of human beings. Although classic risk factors, such as smoking, diabetes mellitus, hyperlipidemia, and hypertension, have been found to predict the occurrence of PAD, there is wide variation in disease manifestation in individuals who have the similar risk profile. Telomere length serves as a potential marker of human aging because of its important features as ‘biological clock’ in genome stability; for example, it decreases progressively with chronological age, varies considerably among people of the similar age, and is strongly linked to inflammation and oxidative stress.

We observed a linear correlation between telomere shortening and chronological age in the current study, and on average the leukocyte telomere length in our PAD patients was comparable to that of control subjects chronologically 5 years older. Our findings lend support to the hypothesis that premature biological aging may contribute to the risk of PAD. Although the exact mechanism explaining the relationship between shorter telomere length and PAD cannot be determined from our study alone, there are several plausible explanations. Decreased telomere length in circulating leukocytes mirrors the replicative history of bone marrow-derived endothelial progenitor cells which have been considered as important agents of vascular repair [43]. Telomere shortening-induced endothelial cell senescence has also been reported in human atherosclerotic plaque lesions [11]. In addition to the effects on vascular repair and endothelial progenitor cell function, telomere shortening can increase the level of circulating pro-inflammatory cytokines and oxidative burden, which are central to the pathogenesis of atherosclerosis and arterial stiffness. Indeed, epidemiological studies have shown that telomere length shortening may be used as a potential marker for age-related diseases [44], most notably coronary heart disease [31], [32].

Of the telomerase-associated pathway genes that encode proteins for the telomere-specific nucleoprotein complex, telomerase reverse transcriptase (*hTERT*) is the rate-limiting catalytic subunit and a major determinant of telomerase activity [45]. *hTERT* expression is strictly regulated at the transcription machinery, and thus it may be speculated that sequence changes in the *hTERT* promoter region interfere with the activation/depression of *hTERT* gene and are associated with the inter-individual variation of telomere length. In the present study, we found that a loss-of-function haplotype C-C (rs2735940 and rs2853669) within the promoter region of the *hTERT* gene is associated with shorter telomere length and increased risk for the development of atherosclerotic PAD. The biological relevance of these observations is further supported by our results in *vivo* luciferase activity assays which showed a lower transcription power for the C-C haplotype.

Genetic variant rs2853669 is located at 190 bp upstream the *hTERT* transcription starting site, residing within a specific binding site for Ets2, which has been suggested to be a positive regulator of the *hTERT* gene and to be required for telomerase activation [38]. A transition from T to C of rs2853669 results in the damage of Ets2 core binding sequence ‘GGAA/T’, which may affect the binding ability of Ets2 and therefore the transcriptional efficiency and expression level of the *hTERT* gene. Consistent with our study, a decreased telomerase activity has been related to the rs2853669C allele in a recent study on non-small cell lung cancer [38]. In addition, the sequence containing rs2853669T/C also partly overlapped with the binding site of transcriptional factor c-Myc which plays a critical role in the *hTERT* gene expression [37], [38]. It is reasonable to speculate that an interaction mechanism exists, which determines the Ets-Myc overall effect at this described polymorphism site. Our experiments in *vivo* luciferase assays further showed that transcription power of the *hTERT* gene was significantly enhanced with the co-expression of Ets2 and c-Myc compared with the presence of either Ets2 or c-Myc. Recent evidence also supports that Ets2 transcription factor probably play as a positive regulator of *hTERT* gene, especially when c-Myc proteins are also expressed [39]. The other variant rs2735940T/C is located at 1327 bp upstream the *hTERT* transcription starting site and its relations with telomere shortening and coronary artery disease are not consistent in various studies [27]–[29]. Although our data did not observe the single-locus association between rs2735940 and telomere length or atherosclerotic PAD, haplotype analysis showed that subjects carrying both rs2735940C and rs2853669C have shorter telomere length and higher prevalence of PAD compared with those individuals with the wild-type haplotype.

Several limitations should be considered. First, not all variants at the *hTERT* gene were assessed in this study, complete sequencing will be necessary for systematic identification of potentially causative mutations. Second, leukocyte telomere lengths were measured from the DNA from patients after diagnosis of PAD in the present study, which in turn could have already been affected by disease progression; therefore, more prospective studies in various populations are needed. Third, technical challenges further complicate the measurement of telomere length. While the quantitative PCR-based assay is the most economical, high-throughput method for telomere length measurements in large epidemiologic studies [31], [32], [36], values are relative representations of the average telomere length. However, the PCR-based method has been previously shown to be highly consistent with classic Southern blotting [31], [32], which suggests that assessment of mean telomere length using the PCR-based method is reliable. The range of T/S ratio in the present study is relatively high than other publications using the similar quantitative PCR method. Most publications used one studied sample as the calibrator sample of telomere length measurement [2], and thus commonly available standards were lack. Here, the present study used the human embryonic kidney 293 cell line as standard, which represent a well-characterized standard to gain comparable measures between studies [46]. More replication studies are further needed.

A number of suggestive genetic loci for telomere length in humans have been identified through family-based linkage studies [19]–[21] and genome-wide association analyses [22]–[26]. Although the genome-wide association studies have been spectacularly successful so far in complex traits, such as myocardial infarction and diabetic mellitus, it is an open question whether we will ultimately be able to identify most of the genetic variation that accounts for common diseases using this approach. For example, only the *TERC* locus, encoding the RNA template component of telomerase, has been identified and replicated in multiple independent populations for its association with telomere length [23]–[25], [47]; however, it accounts for no more than 1% of variation in telomere length [24], and no associations with atherosclerotic vascular diseases were observed [27].

## Conclusions

Aging is a major risk factor for atherosclerotic PAD. Our study showed that shorter leukocyte telomere length is significantly associated with increased risk for the development of PAD, independent of those conventional vascular risk factors. Subjects with the at-risk C-C haplotype (rs2735940 and rs2853669) of the *hTERT* gene (a major determinant of telomerase activity), have shorter telomere length and a higher prevalence of PAD. These results are further supported by functional analysis that the C-C haplotype affects the transcriptional ability of the *hTERT* gene. These observations support the hypothesis that telomere shortening can be used as a prognostic marker for age-related atherosclerotic PAD. Nevertheless, further studies in larger samples are needed to support our intriguing finding.

## Supporting Information

File S1 (PDF)

File S2 Table S1. Primers used for PCR of variants at the *hTERT* gene. Table S2. Primers used for constructing the plasmids. Table S3. Distribution of clinical characteristics by tertiles of leukocyte telomere length in PAD patients and control subjects*. Table S4. Sensitivity analysis for risk of PAD in different tertiles of leukocyte telomere length in the subgroup without cardiovascular disease history (n = 1322). Table S5. Partial spearman correlation coefficients between telomere length and metabolic and anthropometric factors in control subjects*. Table S6. Multivariate-adjusted leukocyte telomere length (relative T/S ratio) according to *hTERT* genotypes*.
(PDF)

File S3 Figure S1. Standard curves for telomere length (A) and the single gene *β-globin* copy (B). Figure S2. Genetic variants in the promoter region of *hTERT* gene. Figure S3. Distribution of relative T/S ratio of leukocyte telomere length in cases and control subjects. Figure S4. Telomere length as a function of age in cases and control subjects. Figure S5. Association between telomere length and the risk of peripheral arterial disease in various subgroups.
(PDF)

## References

[1] Criqui MH, Langer RD, Fronek A, Feigelson HS, Klauber MR, et al (1992) Mortality over a period of 10 years in patients with peripheral arterial disease. N Engl J Med 326: 381–386. 1729621 10.1056/NEJM199202063260605

[2] Zheng ZJ, Sharrett AR, Chambless LE, Rosamond WD, Nieto FJ, et al (1997) Associations of ankle-brachial index with clinical coronary heart disease, stroke and preclinical carotid and popliteal atherosclerosis: the Atherosclerosis Risk in Communities (ARIC) Study. Atherosclerosis 131: 115–125. 9180252 10.1016/s0021-9150(97)06089-9

[3] Newman AB, Shemanski L, Manolio TA, Cushman M, Mittelmark M, et al (1999) Ankle-arm index as a predictor of cardiovascular disease and mortality in the Cardiovascular Health Study. The Cardiovascular Health Study Group. Arterioscler Thromb Vasc Biol 19: 538–545. 10073955 10.1161/01.atv.19.3.538

[4] Hooi JD, Kester AD, Stoffers HE, Rinkens PE, Knottnerus JA, et al (2004) Asymptomatic peripheral arterial occlusive disease predicted cardiovascular morbidity and mortality in a 7-year follow-up study. J Clin Epidemiol 57: 294–300. 15066690 10.1016/j.jclinepi.2003.09.003

[5] Belch JJ, Topol EJ, Agnelli G, Bertrand M, Califf RM, et al (2003) Critical issues in peripheral arterial disease detection and management: a call to action. Arch Intern Med 163: 884–892. 12719196 10.1001/archinte.163.8.884

[6] Brevetti G, Oliva G, Silvestro A, Scopacasa F, Chiariello M (2004) Prevalence, risk factors and cardiovascular comorbidity of symptomatic peripheral arterial disease in Italy. Atherosclerosis 175: 131–138. 15186957 10.1016/j.atherosclerosis.2004.03.009

[7] Hayoz D, Bounameaux H, Canova CR (2005) Swiss Atherothrombosis Survey: a field report on the occurrence of symptomatic and asymptomatic peripheral arterial disease. J Intern Med 258: 238–243. 16115297 10.1111/j.1365-2796.2005.01536.x

[8] Hasimu B, Li J, Nakayama T, Yu J, Yang J, et al (2006) Ankle brachial index as a marker of atherosclerosis in Chinese patients with high cardiovascular risk. Hypertens Res 29: 23–28. 16715650 10.1291/hypres.29.23

[9] Yang X, Sun K, Zhang W, Wu H, Zhang H, et al (2007) Prevalence of and risk factors for peripheral arterial disease in the patients with hypertension among Han Chinese. J Vasc Surg 46: 296–302. 17600667 10.1016/j.jvs.2007.03.034

[10] Blackburn EH (1991) Structure and function of telomeres. Nature 350: 569–573. 1708110 10.1038/350569a0

[11] Minamino T, Miyauchi H, Yoshida T, Ishida Y, Yoshida H, et al (2002) Endothelial cell senescence in human atherosclerosis: Role of telomere in endothelial dysfunction. Circulation 105: 1541–1544. 11927518 10.1161/01.cir.0000013836.85741.17

[12] Nakamura TM, Morin GB, Chapman KB, Weinrich SL, Andrews WH, et al (1997) Telomerase catalytic subunit homologs from fission yeast and human. Science 277: 955–959. 9252327 10.1126/science.277.5328.955

[13] Kilian A, Bowtell DD, Abud HE, Hime GR, Venter DJ, et al (1997) Isolation of a candidate human telomerase catalytic subunit gene, which reveals complex splicing patterns in different cell types. Hum Mol Genet 6: 2011–2019. 9328464 10.1093/hmg/6.12.2011

[14] Ducrest AL, Szutorisz H, Lingner J, Nabholz M (2002) Regulation of the human telomerase reverse transcriptase gene. Oncogene 21: 541–552. 11850779 10.1038/sj.onc.1205081

[15] Yang J, Chang E, Cherry AM, Bangs CD, Oei Y, et al (1999) Human endothelial cell life extension by telomerase expression. J Biol Chem 274: 26141–26148. 10473565 10.1074/jbc.274.37.26141

[16] O'Hare MJ, Bond J, Clarke C, Takeuchi Y, Atherton AJ, et al (2001) Conditional immortalization of freshly isolated human mammary fibroblasts and endothelial cells. Proc Natl Acad Sci U S A 98: 646–651. 11209060 10.1073/pnas.98.2.646 PMC14642

[17] Lansdorp PM, Verwoerd NP, van de Rijke FM, Dragowska V, Little MT, et al (1996) Heterogeneity in telomere length of human chromosomes. Hum Mol Genet 5: 685–691. 8733138 10.1093/hmg/5.5.685

[18] Bischoff C, Graakjaer J, Petersen HC, Hjelmborg JB, Vaupel JW, et al (2005) The heritability of telomere length among the elderly and oldest-old. Twin Res Hum Genet 8: 433–439. 16212832 10.1375/183242705774310141

[19] Vasa-Nicotera M, Brouilette S, Mangino M, Thompson JR, Braund P, et al (2005) Mapping of a major locus that determines telomere length in humans. Am J Hum Genet 76: 147–151. 15520935 10.1086/426734 PMC1196417

[20] Andrew T, Aviv A, Falchi M, Surdulescu GL, Gardner JP, et al (2006) Mapping genetic loci that determine leukocyte telomere length in a large sample of unselected female sibling pairs. Am J Hum Genet 2006 78: 480–486. 16400618 10.1086/500052 PMC1380290

[21] Mangino M, Brouilette S, Braund P, Tirmizi N, Vasa-Nicotera M, et al (2008) A regulatory SNP of the BICD1 gene contributes to telomere length variation in humans. Hum Mol Genet 17: 2518–2523. 18487243 10.1093/hmg/ddn152

[22] Mangino M, Richards JB, Soranzo N, Zhai G, Aviv A, et al (2009) A genome-wide association study identifies a novel locus on chromosome 18q12.2 influencing white cell telomere length. J Med Genet 46: 451–454. 19359265 10.1136/jmg.2008.064956 PMC2696823

[23] Codd V, Mangino M, van der Harst P, Braund PS, Kaiser M, et al (2010) Common variants near terc are associated with mean telomere length. Nat Genet 42: 197–199. 20139977 10.1038/ng.532 PMC3773906

[24] Prescott J, Kraft P, Chasman DI, Savage SA, Mirabello L, et al (2011) Genome-wide association study of relative telomere length. PLoS One 6: e19635. 21573004 10.1371/journal.pone.0019635 PMC3091863

[25] Levy D, Neuhausen SL, Hunt SC, Kimura M, Hwang SJ, et al (2010) Genome-wide association identifies OBFC1 as a locus involved in human leukocyte telomere biology. Proc Natl Acad Sci USA 107: 9293–9238. 20421499 10.1073/pnas.0911494107 PMC2889047

[26] Gu J, Chen M, Shete S, Amos CI, Kamat A, et al (2011) A genome-wide association study identifies a locus on chromosome 14q21 as a predictor of leukocyte telomere length and as a marker of susceptibility for bladder cancer. Cancer Prev Res 4: 514–521. 10.1158/1940-6207.CAPR-11-0063 PMC3076128 21460395

[27] Zee RY, Ridker PM, Chasman DI (2011) Genetic variants in eleven telomere-associated genes and the risk of incident cardio/cerebrovascular disease: The Women's Genome Health Study. Clin Chim Acta 412: 199–202. 20937264 10.1016/j.cca.2010.10.003 PMC3012433

[28] Matsubara Y, Murata M, Watanabe K, Saito I, Miyaki K, et al (2006) Coronary artery disease and a functional polymorphism of hTERT. Biochem Biophys Res Commun 348: 669–672. 16890917 10.1016/j.bbrc.2006.07.103

[29] Nordfjall K, Osterman P, Melander O, Nilsson P, Roos G (2007) hTERT (−1327)T/C polymorphism is not associated with age-related telomere attrition in peripheral blood. Biochem Biophys Res Commun 358: 215–218. 17481586 10.1016/j.bbrc.2007.04.099

[30] Soerensen M, Thinggaard M, Nygaard M, Dato S, Tan Q, et al (2012) Genetic variation in TERT and TERC and human leukocyte telomere length and longevity: a cross-sectional and longitudinal analysis. Aging Cell 11: 223–227. 22136229 10.1111/j.1474-9726.2011.00775.x PMC3303949

[31] Samani NJ, Boultby R, Butler R, Thompson JR, Goodall AH (2001) Telomere shortening in atherosclerosis. Lancet 358: 472–473. 11513915 10.1016/S0140-6736(01)05633-1

[32] Brouilette SW, Moore JS, McMahon AD, Thompson JR, Ford I, et al (2007) Telomere length, risk of coronary heart disease, and statin treatment in the West of Scotland Primary Prevention Study: a nested casecontrol study. Lancet 369: 107–114. 17223473 10.1016/S0140-6736(07)60071-3

[33] Knowles JW, Assimes TL, Li J, Quertermous T, Cooke JP (2007) Genetic susceptibility to peripheral arterial disease: a dark corner in vascular biology. Arterioscler Thromb Vasc Biol 27: 2068–2078. 17656669 10.1161/01.ATV.0000282199.66398.8c PMC4321902

[34] McDermott MM, Lloyd-Jones DM (2009) The role of biomarkers and genetics in peripheral arterial disease. J Am Coll Cardiol 54: 1228–1237. 19778662 10.1016/j.jacc.2009.04.081

[35] Greenland P, Abrams J, Aurigemma GP, Bond MG, Clark LT, et al (2000) Prevention Conference V: Beyond secondary prevention: identifying the high-risk patient for primary prevention: noninvasive tests of atherosclerotic burden: Writing Group III. Circulation 101: E16–22. 10618318 10.1161/01.cir.101.1.e16

[36] Cawthon RM (2002) Telomere measurement by quantitative PCR. Nucleic Acids Res 30: e47. 12000852 10.1093/nar/30.10.e47 PMC115301

[37] Horikawa I, Cable PL, Afshari C, Barrett JC (1999) Cloning and characterization of the promoter region of human telomerase reverse transcriptase gene. Cancer Res 59: 826–830. 10029071

[38] Hsu CP, Hsu NY, Lee LW, Ko JL (2006) Ets2 binding site single nucleotide polymorphism at the hTERT gene promoter: effect on telomerase expression and telomere length maintenance in non-small cell lung cancer. Eur J Cancer 42: 1466e74. 16737810 10.1016/j.ejca.2006.02.014

[39] Takakura M, Kyo S, Kanaya T, Hirano H, Takeda J, et al (1999) Cloning of human telomerase catalytic subunit (hTERT) gene promoter and identification of proximal core promoter sequences essential for transcriptional activation in immortalized and cancer cells. Cancer Res 59: 551–557. 9973199

[40] Simes RJ (1986) An improved Bonferroni procedure for multiple tests of significance. Biometrika 73: 751–754.

[41] Tregouet DA, Escolano S, Tiret L, Mallet A, Golmard JL (2004) A new algorithm for haplotype-based association analysis: the Stochastic-EM algorithm. Ann Hum Genet 68: 165–177. 15008795 10.1046/j.1529-8817.2003.00085.x

[42] Gauderman WJ, Morrison JM (2006) Quanto 1.1: a computer program for power and sample size calculations for genetic epidemiology studies (http://hydra.usc.edu/gxe).

[43] Ju Z, Jiang H, Jaworski M, Rathinam C, Gompf A, et al (2007) Telomere dysfunction induces environmental alterations limiting hematopoietic stem cell function and engraftment. Nat Med 13: 742–747. 17486088 10.1038/nm1578

[44] Calado RT, Young NS (2009) Telomere diseases. N Engl J Med 361: 2353–2365. 20007561 10.1056/NEJMra0903373 PMC3401586

[45] Blackburn EH, Greider CW, Szostak JW (2006) Telomeres and telomerase: the path from maize, Tetrahymena and yeast to human cancer and aging. Nat Med 12: 1133–1138. 17024208 10.1038/nm1006-1133

[46] Fehrer C, Voglauer R, Wieser M, Pfister G, Brunauer R, et al (2006) Techniques in gerontology: Cell lines as standards for telomere length and telomerase activity assessment. Exp Gerontol 41: 648–651. 16677791 10.1016/j.exger.2006.03.016

[47] Shen Q, Zhang Z, Yu L, Cao L, Zhou D, et al (2011) Common variants near TERC are associated with leukocyte telomere length in the Chinese Han population. Eur J Hum Genet 19: 721–723. 21304559 10.1038/ejhg.2011.4 PMC3110055

