# Supplementary material for: Functional Haplotypes of the hTERT Gene, Leukocyte Telomere Length Shortening, and the Risk of Peripheral Arterial Disease
Source: PLoS One. 2012 Oct 17;7(10):e47029. doi: 10.1371/journal.pone.0047029 (PMC3474805; doi:10.1371/journal.pone.0047029)
Supplement: File S1 — (PDF) [file pone.0047029.s001.pdf]

Full title: Functional haplotypes of the hTERT gene, leukocyte telomere length shortening, and the risk of peripheral arterial disease

Supplemental file S1

ABI Measurement

The ABI was determined from Doppler-derived measurements of systolic blood pressure (SBP) at the brachial and ankle arteries. Briefly, after a 5-minute rest, appropriate size cuffs were placed on each arm and ankle, and a Doppler ultrasonic device (ES-101EX, HADECO, 8 MHz probe, Kawasaki, Japan) was used to detect each pulse. The cuff was inflated to 20 mmHg above the palpated SBP and deflated at 2 mmHg per pulse. The first reappearance of the pulse was recorded as the SBP. To calculate ABI, the SBP at each ankle site was divided by the higher of two brachial blood pressures. The lowest ratio was designated as the ABI [1].

Leukocyte Telomere Length Assay

Relative mean leukocyte telomere length was determined with a quantitative real-time polymerase chain reaction (PCR)-based technique that compares telomere repeat copy number (T) to single-copy gene copy number (S) (T/S ratio) in a given sample [2]. All PCRs were performed on the Bio-Rad DNA Engine Opticon 2 Real-time PCR Detector (Bio-Rad Ltd, Hercules, CA, USA).

In brief, two master mixes of PCR reagents were prepared, one for the telomere reaction and one for the single-copy gene reaction (*β-globin* gene on chromosome 11p15.5). The final concentrations of reagents in the PCR (20 μl) were 0.2 × Sybr Green I, 0.2 mM each dNTP, 2.5 mM DTT, l% DMSO, 0.2 μl of 10× buffer, and 0.5 U Hotstart *Taq* DNA polymerase (TaKaRa), 200 nM primer concentrations, and 3 ng/μl of genomic DNA. The primer sequences were as follows:

telomere forward 5'- CGGTTTGTTTGGGTTTGGGTTTGGGTTTGGGTTTGGGTT-3';
telomere reverse 5'- GGCTTGCCTTACCCTTACCCTTACCCTTACCCTTACCCT-3';
β-globin forward 5'- GCTTCTGACACAACTGTGTTCACTAGC-3';
β-globin reverse 5'- CACCAACTTCATCCACGTTCACC-3'.

The thermal cycling profile began with 95°C incubation for 5 min to activate the *Hotstart* DNA polymerase. For telomere PCR, there followed 33 cycles of 95°C for 15 sec, 54.3°C for 1 min; for *β-globin* PCR, there followed 37 cycles of 95°C for 15 sec, 56°C for 30 sec, 72°C for 30 sec. Melting curve analysis was performed on every run to verify the PCR products.

All samples for both the telomere and single-copy gene amplifications were done in duplicate in 96-well plates. When the duplicate T/S value and the initial value varied by more than 7%, the sample was run for a third time, and the 2 closest values were used to calculate the mean. The human embryonic kidney 293 (HEK293S) cell line was used as standards for the measurement of mean telomere length. A dilution series (1.56 to 100.00 ng; 2-fold dilution; 7 points) using genomic DNA derived from the HEK293S cell line were included

with each 96-well plate for the telomere and the *β-globin* PCRs. The 25-ng standard curve point was used as the reference sample. The slope of the standard curve for the telomere and *β*-globin reactions was -0.22 and -0.35, respectively, and the linear correlation coefficient (R^2) value for both reactions were >0.99 (Supplemental Figure S1). The average inter-plate coefficient of variability was 6.6% for telomere assays and 4.8% for *β-globin* assays. Test samples with threshold cycle numbers that fell outside the range defined by the standard curves were rerun at different concentrations to ensure that they were amplified within the linear range. As part of routine quality control, 10% of the samples were blinded reproducibility samples. All measurements were performed by laboratory personnel blinded to case-control status and the outcome assessment.

References:

1. Greenland P, Abrams J, Aurigemma GP, Bond MG, Clark LT, et al. (2000) Prevention Conference V: Beyond secondary prevention: identifying the high-risk patient for primary prevention: noninvasive tests of atherosclerotic burden: Writing Group III. Circulation 101: E16-22.
2. Cawthon RM. (2002) Telomere measurement by quantitative PCR. *Nucleic Acids Res*. 30(10):e 47.
