# Supplementary material for: Functional Haplotypes of the hTERT Gene, Leukocyte Telomere Length Shortening, and the Risk of Peripheral Arterial Disease
Source: PLoS One. 2012 Oct 17;7(10):e47029. doi: 10.1371/journal.pone.0047029 (PMC3474805; doi:10.1371/journal.pone.0047029)
Supplement: File S2 — Table S1. Primers used for PCR of variants at the hTERT gene. Table S2. Primers used for constructing the plasmids. Table S3. Distribution of clinical characteristics by tertiles of leukocyte telomere length in PAD patients and control subjects*. Table S4. Sensitivity analysis for risk of PAD in different tertiles of leukocyte telomere length in the subgroup without cardiovascular disease history (n = 1322). Table S5. Partial spearman correlation coefficients between telomere length and metabolic and anthropometric factors in control subjects*. Table S6. Multivariate-adjusted leukocyte telomere length (relative T/S ratio) according to hTERT genotypes*. (PDF) [file pone.0047029.s002.pdf]

Full title: Functional haplotypes of the hTERT gene, leukocyte telomere length shortening, and the risk of peripheral arterial disease

Supplemental file S2

Table S1. Primers used for PCR of variants at the *hTERT* gene

Table S2. Primers used for constructing the plasmids

Table S3. Distribution of clinical characteristics by tertiles of leukocyte telomere length in PAD patients and control subjects*

Table S4. Sensitivity analysis for risk of PAD in different tertiles of leukocyte telomere length in the subgroup without cardiovascular disease history (n=1322)

Table S5. Partial spearman correlation coefficients between telomere length and metabolic and anthropometric factors in control subjects*

Table S6. Multivariate-adjusted leukocyte telomere length (relative T/S ratio) according to *hTERT* genotypes*

Table S1. Primers used for PCR of variants at the *hTERT* gene

Variants	Primer sequences	PCR product length (bp)	Annealing temperature (°C)	Restriction enzyme
rs2735940 (-1327T/C)	F: 5’ GCAGAGAACCAGTGTAAGCTACAACTT3’	180	54	PstI
	R: 5’ GGTTAGCCTCGTCTTGTAAATACTTAGGACTGC 3’			
rs2853669 (-190T/C)	F: 5’ GCGGGCACAGACGCCCAGGACCGAGCT 3’	184	69	SacI
	R: 5’ GCGGAAAGGAAGGGGAGGGGCTGGGA 3’			

PCR, polymerase chain reaction; F: Forward; R: Reverse.

Table S2. Primers used for constructing the plasmids

Plasmids	Genbank accession no.	Primer sequences
pGL3-rs2853669 of the *hTERT* gene	AF098956	F: 5’GGCCGGTACCTCGGGTTACCCCACAGCCTA3’<br>R: 5’GGCAAGCTTCAGCACCTCGCGGTAGTGGCT3’
pGL3-haplotype of the *hTERT* gene	AF098956	F: 5’CCAAGGTACCCCTTTGCCCTAGTGGCAGAGACA3’<br>R: 5’GGCAAGCTTCAGCACCTCGCGGTAGTGGCT3’
pcDNA3.1-C-Myc	NM_002467.4	F: 5'CTGAAAGCTTATGCCCCTCAACGTTAGCTTCACCA3’<br>R: 5'GTGGGATCCTTACGCACAAGAGTTCCGTAGCTGTT3’
pcDNA3.1-Ets2	NM_005239.5	F: 5'CAGCAAGCTTATGAATGATTTCGGAATCAAGAATATGGA3'<br>R: 5’AATGGATCCTCAGTCCTCCGTGTCGGG3'

Table S3. Distribution of clinical characteristics by tertiles of leukocyte telomere length in PAD patients and control subjects*

	Cases with PAD (n=485)				Control subjects (n= 970)			
Characteristics	Highest tertile (n=79)	Middle tertile (n=147)	Lowest tertile (n=259)	*P* value†	Highest tertile (n=324)	Middle tertile (n=324)	Lowest tertile (n=322)	*P* value†
Telomere length (relative T/S ratio), Median	3.95	2.36	1.29		4.25	2.42	1.33	
Age, years	58.2 ± 9.6	58.5 ± 9.0	58.8 ± 9.5	0.87	58.5 ± 8.9	58.0 ± 9.4	58.4 ± 9.8	0.81
Male, n (%)	26 (32.9%)	47 (32.0%)	106 (40.9%)	0.14	105 (32.4%)	137 (42.2%)	130 (40.4%)	0.03
Body mass index, kg/m^2	24.5 ± 3.5	25.4 ± 3.6	25.3 ± 3.8	0.16	25.7 ± 3.6	25.6 ± 3.4	25.6 ± 3.8	0.91
Waist-hip ratio	0.86 ± 0.06	0.87 ± 0.06	0.87 ± 0.06	0.28	0.86 ± 0.05	0.87 ± 0.06	0.87 ± 0.06	0.17
Systolic BP, mm Hg	166 ± 31	166 ± 26	161 ± 29	0.14	158 ± 28	158 ± 30	158 ± 26	0.98
Diastolic BP, mm Hg	97 ± 13	97 ± 12	95 ± 15	0.22	95 ± 13	94 ± 14	95 ± 12	0.71
Glucose, mmol/L	5.27 ± 1.64	5.86 ± 2.45	5.60 ± 2.16	0.14	5.38 ± 1.75	5.57 ± 2.12	5.51 ± 1.73	0.42
Lipids, mmol/L								
Total cholesterol	5.37 ±1.08	5.63 ± 1.37	5.64 ± 1.27	0.25	5.49 ± 1.10	5.43 ± 1.12	5.46 ± 1.15	0.80
Triglycerides	1.19 (0.83-1.68)	1.39 (0.92-1.89)	1.45 (1.04-2.13)	0.02	1.33 (0.93-1.85)	1.29 (0.94-1.78)	1.30 (0.97-1.79)	0.86
HDL_C	1.59 ±0.30	1.53 ± 0.34	1.58 ± 0.34	0.32	1.56 ±0.33	1.52 ± 0.33	1.56 ± 0.38	0.38
LDL_C	2.99 ± 0.88	3.18 ± 0.97	3.22 ± 1.01	0.21	3.11 ± 0.89	3.12 ± 0.91	3.06 ± 0.90	0.64
Cigarette smoking, n (%)	16 (20.3%)	31 (21.1%)	58 (22.4%)	0.90	49 (15.1%)	59 (18.2%)	70 (21.7%)	0.09
Alcohol intake, n (%)	18 (22.8%)	35 (23.8%)	61 (23.6%)	0.98	56 (17.3%)	67 (20.6%)	56 (17.3%)	0.30
Medical history, n (%)								
Hypertension	51 (64.6%)	106 (72.1%)	189 (73.0%)	0.34	204 (63.0%)	200 (61.5%)	210 (65.2%)	0.62
Diabetes mellitus	1 (2.0%)	10 (6.8%)	10 (3.5%)	0.05	5 (1.5%)	16 (4.9%)	14 (4.3%)	0.05
Cardiovascular disease	15 (19.0%)	30 (20.4%)	58 (22.4%)	0.78	8 (2.5%)	12 (3.7%)	11 (3.4%)	0.65
Medication treatment, n (%)								
Antihypertension %	46 (90.2%)	92 (86.8%)	156 (82.5%)	0.33	170 (83.3%)	163 (81.1%)	174 (82.9%)	0.82
Blood-glucose control, n (%)	3 (75.0%)	8 (80.0%)	7 (100.0%)	0.41	3 (50.0%)	13 (81.3%)	9 (64.3%)	0.32
Lipid-lowing therapy, n (%)	12 (75.0%)	13 (54.2%)	27 (69.2%)	0.33	17 (53.1%)	34 (68.0%)	40 (70.2%)	0.24

PAD indicates peripheral arterial disease; BP, blood pressure; HDL-C, high-density lipoprotein cholesterol; LDL-C, low-density lipoprotein cholesterol; T, telomere repeat copy; S, single-copy gene *globin* copy.

* Telomere length is expressed as a relative telomere/single-copy gene (T/S) ratio. Limits for the tertiles of telomere length were derived from the control group, and relative T/S ratios for tertile cutoffs were <1.87 for lowest tertile, 1.87 to 3.23 for the middle tertile, and >3.23 for highest tertile. Data are given as mean ± SD, numbers (percentage) or medians (interquartile range).

† *P* value was obtained by ANOVA for comparison of continuous variables, the χ^2 test for categorical variables, and the Kruskal-Wallis H test for triglycerides.

Table S4. Sensitivity analysis for risk of PAD in different tertiles of leukocyte telomere length in the subgroup without cardiovascular disease history (n=1322)

	In tertile groups of relative T/S ratio				Per 1-SD decrease in ln-transformed relative T/S ratio	*P*
	Highest tertile (>3.23)	Middle tertile (1.87-3.23)	Lowest tertile (<1.87)	*P* for trend		
Cases with PAD (n=382)	64 (16.8%)	117 (30.6%)	201 (52.6%)			
Control subjects (n= 940)	316 (33.7%)	312 (33.2%)	311 (33.1%)			
Odds ratio(95%CI) *						
Crude model	1.0	1.85 (1.32-2.61)	3.19 (2.31-4.40)	<0.0001	1.71 (1.51-1.94)	<0.0001
Multivariable model I†	1.0	1.89 (1.34-2.66)	3.25 (2.35-4.50)	<0.0001	1.72 (1.51-1.95)	<0.0001
Multivariable model II‡	1.0	1.83 (1.33-2.64)	3.14 (2.27-4.33)	<0.0001	1.70 (1.50-1.93)	<0.0001

PAD indicates peripheral arterial disease; CI, confidence interval.

*Odds ratio and 95%CI were obtained with multivariate conditional logistic regression analysis.

†Model I: Adjustment for body mass index, systolic and diastolic blood pressure, smoking, alcohol intake, fasting glucose, triglycerides, total cholesterol, HDL-C, and LDL-C.

‡Model II: Adjustment for the covariates mentioned above plus diabetes, history of hypertension, and medication treatment.

Table S5. Partial spearman correlation coefficients between telomere length and metabolic and anthropometric factors in control subjects

	Telomere Length (relative T/S ratio) *	
	Beta coefficient	*P*
Body mass index, kg/m^2	0.01	0.60
Waist-hip ratio	-0.04	0.06
Systolic blood pressure, mmHg	-0.03	0.26
Diastolic blood pressure, mmHg	-0.02	0.40
Serum glucose, mmol/L	-0.03	0.24
Serum creatinine, µmol/L	0.03	0.34
Triglycerides, mmol/L	-0.01	0.81
Total cholesterol,	-0.06	0.02
HDL cholesterol, mmol/L	-0.05	0.07
LDL cholesterol, mmol/L	-0.06	0.03

* Ln-transformed relative telomere/single-copy gene (T/S) ratio was used. Correlation coefficients were adjusted for age, gender, smoking status, and alcohol intake.

Table S6. Multivariate-adjusted leukocyte telomere length (relative T/S ratio) according to *hTERT* genotypes

	rs2735940 (-1327T>C)				rs2853669 (-190 T>C)			
	TT	TC	CC	*P*†	TT	TC	CC	*P*†
Controls (n=970)								
N (%)	322 (33.3%)	522 (53.8%)	126 (13.0%)		367 (37.8%)	478 (49.3%)	125 (12.9%)	
Crude model	2.79 ± 0.09	2.71 ± 0.07	2.69 ± 0.14	0.70	2.79 ± 0.08	2.74 ± 0.07	2.55 ± 0.14	0.30
Multivariate-adjusted‡	2.51 ± 0.16	2.42 ± 0.16	2.39 ± 0.20	0.67	2.51 ± 0.16	2.45 ± 0.16	2.24 ± 0.20	0.23
Cases (n=485)								
N (%)	160 (33.0%)	239 (49.3%)	86 (17.7%)		149 (30.7%)	252 (52.0%)	84 (17.3%)	
Crude model	2.15 ± 0.14	2.01 ± 0.14	1.88 ± 0.16	0.13	2.19 ± 0.14	2.04 ± 0.14	1.75 ± 0.16	0.006
Multivariate-adjusted‡	1.97 ± 0.08	1.84 ± 0.07	1.73 ± 0.11	0.18	2.03 ± 0.08	1.86 ± 0.06	1.60 ± 0.11¶	0.006

Data were presented as mean ± S.E.M. S.E.M denotes standard error of the mean.
†General linear models were used to compare means of telomere length across the genotypes.
‡Model adjusted for body mass index, systolic and diastolic blood pressure, smoking, alcohol intake, fasting glucose, triglycerides, total cholesterol, HDL cholesterol, LDL cholesterol, diabetes, history of hypertension, and medication treatment.
¶Significantly different from the TT genotype, *P*=0.005 (Tukey's test).
