# Supplementary material for: Functional Haplotypes of the hTERT Gene, Leukocyte Telomere Length Shortening, and the Risk of Peripheral Arterial Disease
Source: PLoS One. 2012 Oct 17;7(10):e47029. doi: 10.1371/journal.pone.0047029 (PMC3474805; doi:10.1371/journal.pone.0047029)
Supplement: File S3 — Figure S1. Standard curves for telomere length (A) and the single gene β-globin copy (B). Figure S2. Genetic variants in the promoter region of hTERT gene. Figure S3. Distribution of relative T/S ratio of leukocyte telomere length in cases and control subjects. Figure S4. Telomere length as a function of age in cases and control subjects. Figure S5. Association between telomere length and the risk of peripheral arterial disease in various subgroups. (PDF) [file pone.0047029.s003.pdf]

Full title: Functional haplotypes of the hTERT gene, leukocyte telomere length shortening, and the risk of peripheral arterial disease

Supplemental file S3

Figure S1. Standard curves for telomere length (A) and the single gene *β-globin* copy (B)

Figure S2. Genetic variants in the promoter region of *hTERT* gene

Figure S3. Distribution of relative T/S ratio of leukocyte telomere length in cases and control subjects

Figure S4. Telomere length as a function of age in cases and control subjects

Figure S5. Association between telomere length and the risk of peripheral arterial disease in various subgroups

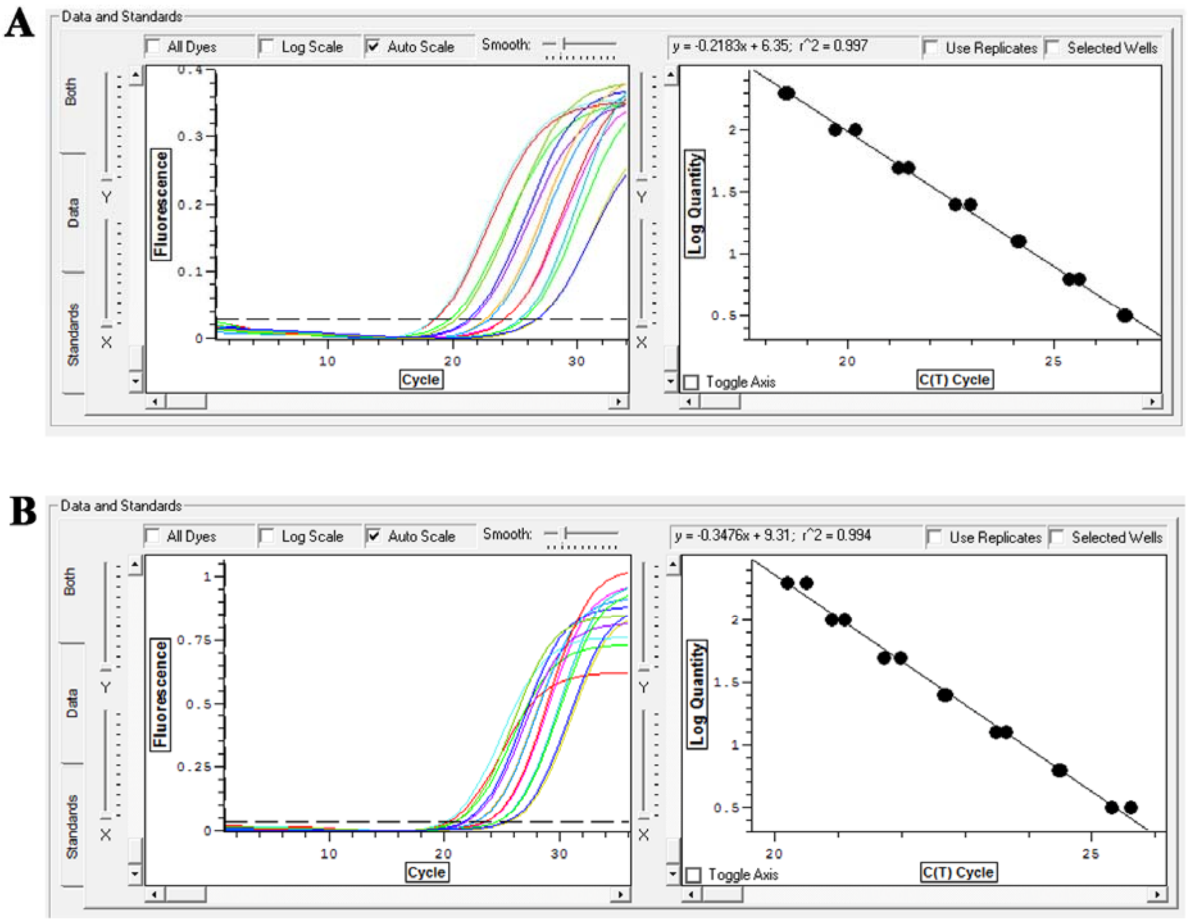

Figure S1. Standard curves for telomere length (A) and the single gene *β-globin* copy (B)

A dilution series (1.56 to 100.00 ng; 2-fold dilution; 7 points) using genomic DNA derived from the HEK293S cell line were produced for the generation of standard curves. The slope of the standard curve for the telomere and *β*-globin reactions was -0.22 and -0.35, respectively, and the linear correlation coefficient (R^2) value for both reactions were >0.99.

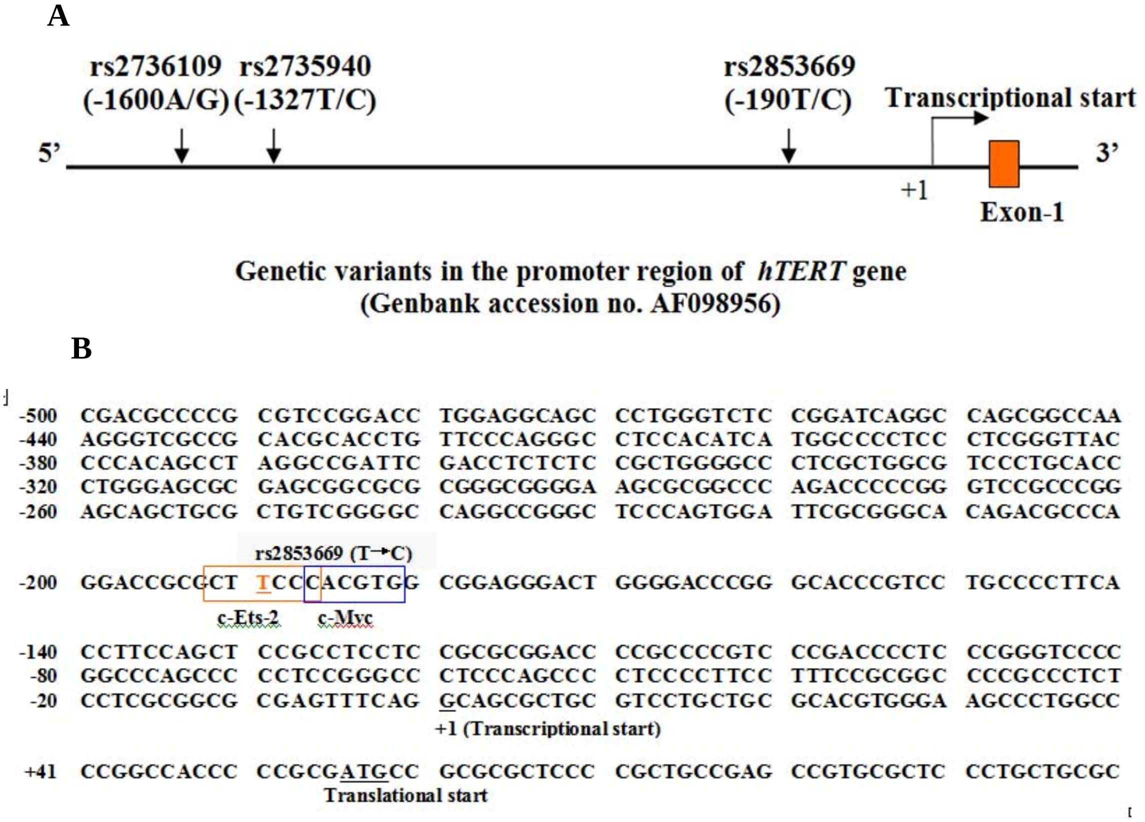

Figure S2. Genetic variants in the promoter region of *hTERT* gene

A. Schematics of genetic variants in the promoter region of the *hTERT* gene (nucleotide numbering from the transcriptional start site as +1);

B. The position of rs2853669 resides within a specific binding site of transcription factor Ets2 and partly overlapped with c-Myc.

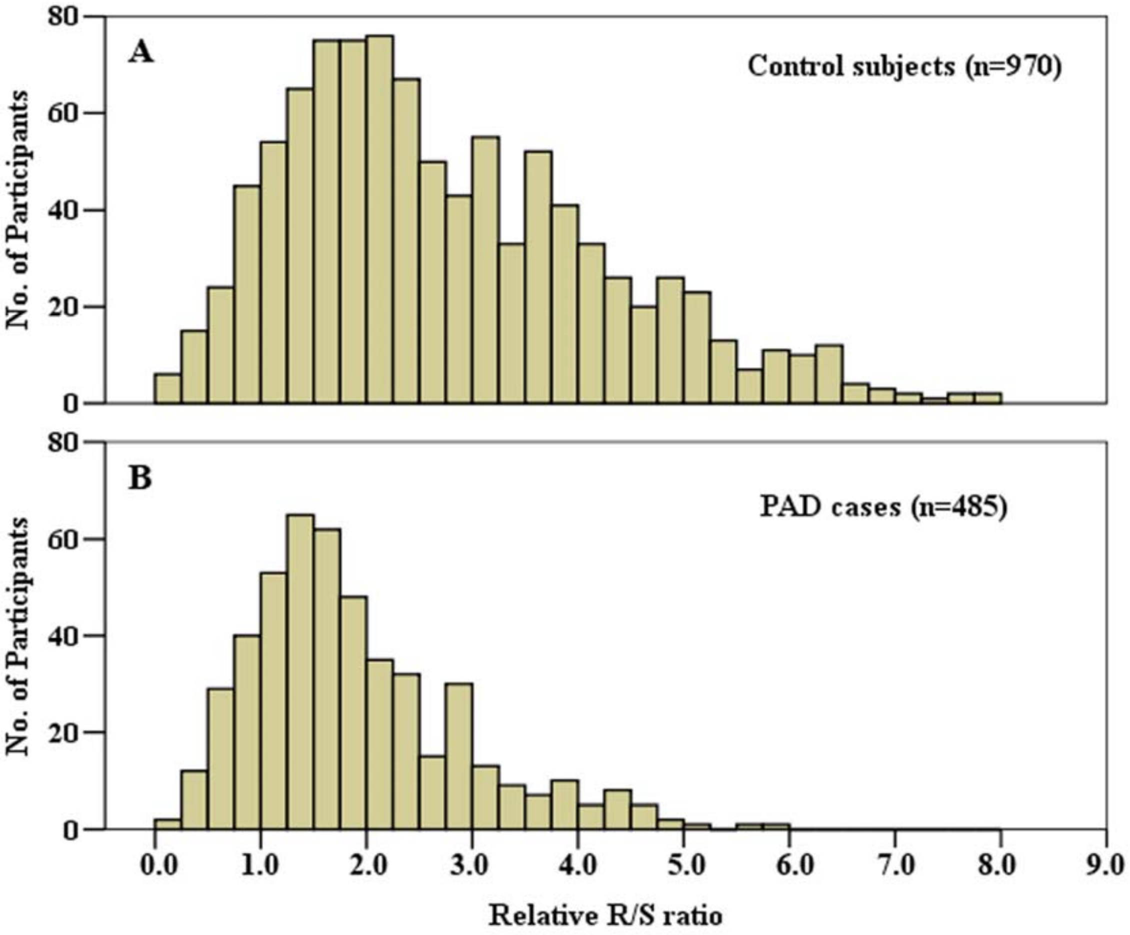

Figure S3. Distribution of relative T/S ratio of leukocyte telomere length in cases and control subjects

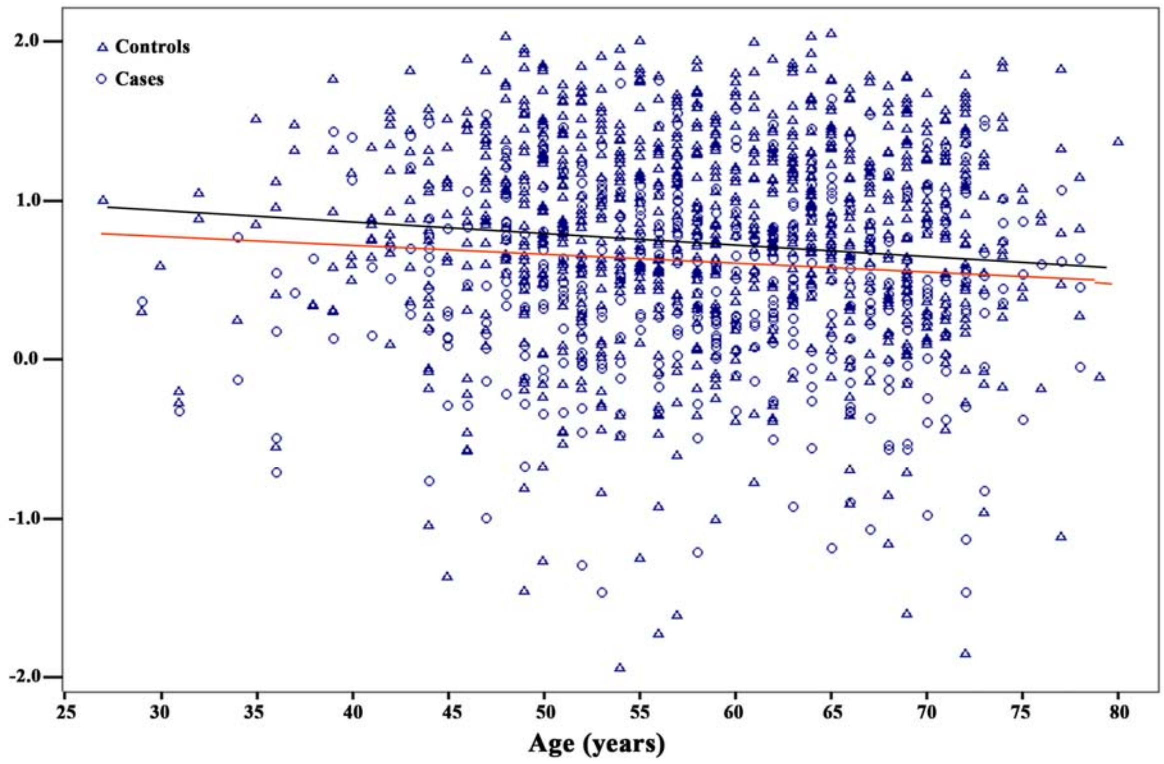

Figure S4. Telomere length as a function of age in cases and control subjects

Controls are shown as blue triangles (n=970) and cases as blue circles (n=485). Telomere length is expressed as the natural log (ln) of relative telomere to single-copy gene (T/S) ratio. Slope of the line indicates the annual telomere-shortening rate in control subjects (coefficient correlation γ = -0.13, P<0.001; black line) and PAD cases (coefficient correlation γ = -0.11, P<0.001; red line), respectively.

	PAD cases (n=485)	Odds ratio (95%CI) Per 1-SD Decrease in -transformed relative T/S ratio
**Gender**		
Male (n=551)	179	1.89 (1.53-2.34)
Female (n=905)	306	1.68 (1.45-1.95)
**Body mass index**		
<25 (n=681)	242	1.74 (1.47-2.07)
≧25 (n=775)	243	1.72 (1.44-2.05)
**Medication**		
No (n=587)	161	1.79 (1.47-2.19)
Yes (n=869)	324	1.69 (1.45-1.97)
**Smoking status**		
Nonsmoker (n=1173)	380	1.77 (1.54-2.02)
Smokers (n=283)	105	1.58 (1.20-2.10)
**Alcohol intake**		
Nondrinker (n=1148)	371	1.70 (1.48-1.94)
Drinkers (n=308)	114	1.88 (1.41-2.50)
**History of hypertension**		
No (n=496)	346	1.62 (1.31-2.00)
Yes (n=960)	139	1.78 (1.53-2.07)
**rs2735940**		
TT (n=483)	160	2.03 (1.60-2.58)
TC (n=761)	239	1.67 (1.43-1.96)
CC (n=212)	86	1.82 (1.35-2.45)
**rs2853669**		
TT (n=516)	149	1.62 (1.32-1.99)
TC (n=731)	252	1.77 (1.4-2.09)
CC (n=209)	84	2.09 (1.50-2.90)
**Age, years**		
25-39 (n=36)	12	2.71 (0.92-7.80)
40-49 (n=220)	71	1.62 (1.20-2.19)
50-59 (n=531)	171	1.56 (1.29-1.89)
60-69 (n=478)	161	2.30 (1.82-2.90)
>70 (n=191)	70	1.73 (1.26-2.37)

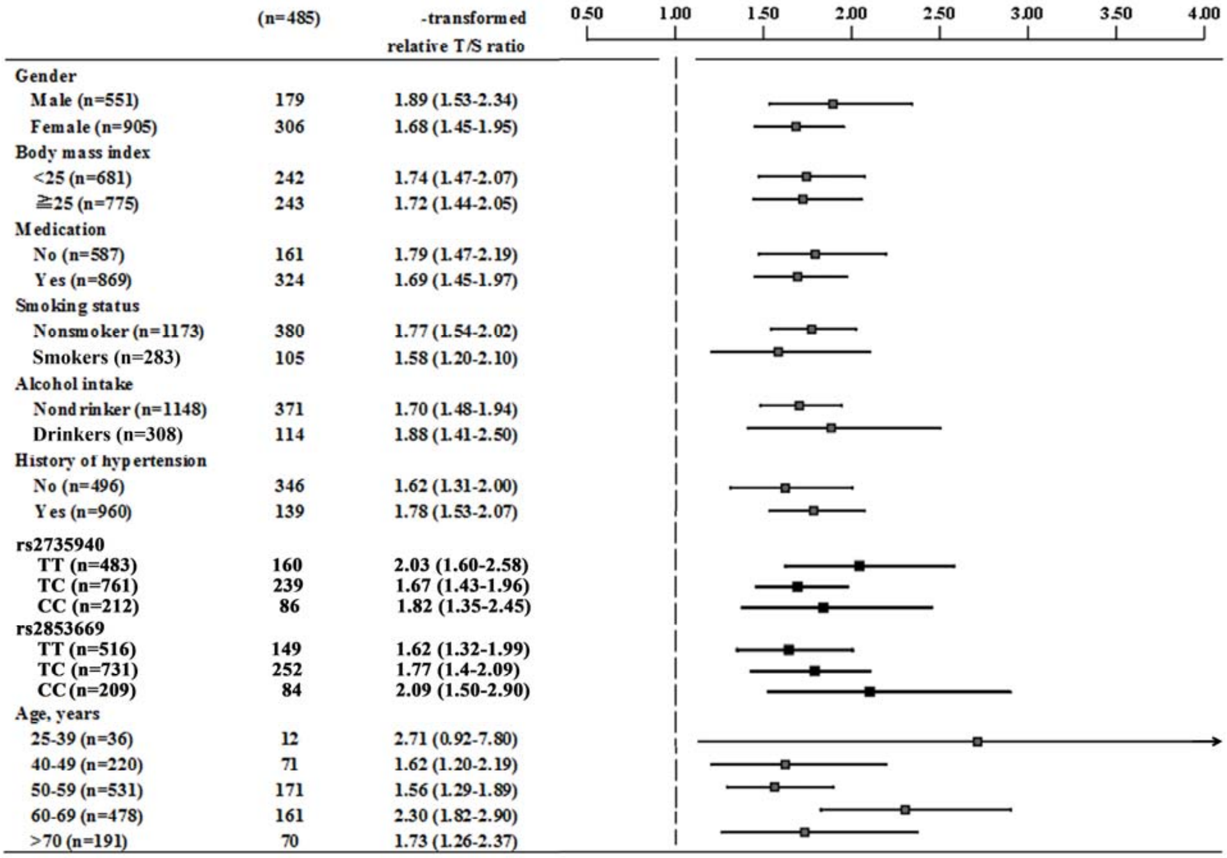

Figure S5. Association between telomere length and the risk of PAD in various subgroups

Odds ratio and 95%CI were obtained with multivariate conditional logistic regression analysis in various subgroups with adjustment age, gender, body mass index, systolic and diastolic blood pressure, smoking, alcohol intake, fasting glucose, triglycerides, total cholesterol, HDL cholesterol, and LDL cholesterol, diabetes, history of hypertension, previous cardiovascular disease, and medication treatment when appropriate.
